# Re-Emergence of Usutu Virus and Spreading of West Nile Virus Neuroinvasive Infections During the 2024 Transmission Season in Croatia

**DOI:** 10.3390/v17060846

**Published:** 2025-06-13

**Authors:** Tatjana Vilibić-Čavlek, Ljubo Barbić, Ana Klobučar, Marko Vucelja, Maja Bogdanić, Dario Sabadi, Marko Kutleša, Branimir Gjurašin, Vladimir Stevanović, Marcela Curman Posavec, Linda Bjedov, Marko Boljfetić, Tonka Jozić-Novinc, Robert Škara, Morana Tomljenović, Željka Hruškar, Mahmoud Al-Mufleh, Tanja Potočnik-Hunjadi, Ivana Rončević, Vladimir Savić

**Affiliations:** 1 Department of Virology, Croatian Institute of Public Health, 10000 Zagreb, Croatia; maja.bogdanic@hzjz.hr (M.B.); zeljka.hruskar@hzjz.hr (Ž.H.); 2 School of Medicine, University of Zagreb, 10000 Zagreb, Croatia; 3 Department of Microbiology and Infectious Diseases with Clinic, Faculty of Veterinary Medicine, University of Zagreb, 10000 Zagreb, Croatia; ljubo.barbic@vef.hr (L.B.); vladostevanovic@gmail.com (V.S.); 4 Department of Epidemiology, Andrija Štampar Teaching Institute of Public Health, 10000 Zagreb, Croatia; ana.klobucar@stampar.hr (A.K.); marcela.curman@stampar.hr (M.C.P.); 5 Department of Forest Protection and Wildlife Management, Faculty of Forestry and Wood Technology, University of Zagreb, 10000 Zagreb, Croatia; mvucelja@sumfak.unizg.hr (M.V.); lbjedov@sumfak.unizg.hr (L.B.); mboljfetic@sumfak.unizg.hr (M.B.); 6 Department of Infectious Diseases, Clinical Hospital Center Osijek, 31000 Osijek, Croatia; dariocroatia@gmail.com; 7 Faculty of Medicine, Josip Juraj Strossmayer University of Osijek, 31000 Osijek, Croatia; 8 Department of Intensive Care Medicine and Neuroinfectology, University Hospital for Infectious Diseases “Dr. Fran Mihaljević”, 10000 Zagreb, Croatia; mkutlesa@bfm.hr (M.K.); bgjurasin@bfm.hr (B.G.); 9 Department of Infectious Diseases, General Hospital “Dr. Ivo Pedišić”, 44000 Sisak, Croatia; tonkajn@gmail.com (T.J.-N.); infektologija@obs.hr (R.Š.); 10 Department of Epidemiology, Teaching Institute of Public Health of the Primorje-Gorski Kotar County, 51000 Rijeka, Croatia; tomljenovicmorana@gmail.com; 11 Department of Social Medicine and Epidemiology, Faculty of Medicine, University of Rijeka, 51000 Rijeka, Croatia; 12 Department of Infectious Diseases, County Hospital Čakovec, 40000 Čakovec, Croatia; mahmoud.almufleh@gmail.com; 13 Department of Infectious Diseases, General Hospital Varaždin, 42000 Varaždin, Croatia; tanja.potocnik.h@gmail.com; 14 Poultry Center, Croatian Veterinary Institute, 10000 Zagreb, Croatia; roncevic@veinst.hr

**Keywords:** West Nile virus, Usutu virus, tick-borne encephalitis virus, neuroinvasive infections, Croatia

## Abstract

Neuroinvasive arboviruses such as tick-borne encephalitis virus (TBEV), West Nile virus (WNV), Usutu virus (USUV), and Toscana virus (TOSV) have (re-)emerged with increasing incidence and geographic range. We analyzed the epidemiology of arboviral infections in Croatia during the 2024 transmission season. A total of 154 patients with neuroinvasive diseases (NID), 1596 horses, 69 dead birds, and 7726 mosquitoes were tested. Viral RNA was detected using RT-qPCR. IgM/IgG-specific antibodies were detected using commercial ELISA or IFA, with confirmation of cross-reactive samples by virus neutralization test. RT-qPCR-positive samples were Sanger sequenced. Arboviral etiology was confirmed in 33/21.42% of patients with NID. WNV was most frequently detected (17/11.03%), followed by TBEV (10/6.49%), USUV (5/3.24%), and TOSV (1/0.64%). WNV infections were reported in regions previously known as endemic, while in one continental county, WNV was recorded for the first time. USUV infections re-emerged after a six-year absence. In addition to human cases, acute WNV infections were recorded in 11/395 (2.78%) of horses and two dead crows. WNV IgG seropositivity was detected in 276/1168 (23.63%) and TBEV IgG seropositivity in 68/428 (15.88%) horses. None of the tested mosquito pools were positive for WNV and USUV RNA. Phylogenetic analysis showed the circulation of WNV lineage 2 and Usutu Europe 2 lineage. Climate conditions in 2024 in Croatia were classified as extremely warm, which could, at least in part, impact the quite intense arboviral season. The spreading of flaviviruses in Croatia highlights the need for continuous surveillance in humans, animals, and vectors (“One Health”).

## 1. Introduction

Arboviruses represent an emerging public health threat in many regions of the world. The combination of factors, including global travel and trade, climate change, and vectors’ adaptation and spread, further increases the risk of arboviral infections both within and outside of endemic areas [1,2]. Some studies have shown that climate change has been an important factor contributing to the increased risk of WNV circulation in Europe. Temperature is a major driver of mosquito-borne disease transmission. Temperature affects the mosquito biting rates and shortens the extrinsic incubation period in mosquitoes [3]. Similarly, tick-borne encephalitis (TBE) cases have risen geographically during the past five years [4,5]. Tick development and activity, as well as their latitudinal and altitudinal range limit, are significantly impacted by air temperature [6].

Although most arboviral infections are asymptomatic or mild, some patients, especially the elderly and immunocompromised, develop severe neuroinvasive disease (NID) such as meningitis, encephalitis, or myelitis. Among neuroinvasive arboviruses, flaviviruses such as tick-borne encephalitis (TBEV) and West Nile virus (WNV) are the most commonly detected. TBEV is the most widely distributed tick-borne arbovirus in Europe; however, endemic areas also include northern and eastern Asia [7]. WNV is the most prevalent mosquito-borne viral zoonosis in southern, western, and Eastern Europe. Human Usutu virus (USUV) infections are still underreported despite a wide distribution of this arbovirus in different animal species [8]. Toscana virus (TOSV) is an emerging bunyavirus with an increasing geographic range throughout the Mediterranean Basin, Europe, and the Middle East [9,10].

There are three main TBEV subtypes: European (TBEV-Eu), Far-Eastern (TBEV-FE), and Siberian (TBEV-Sib), with more recently described Baikalian and Himalayan subtypes. TBEV-Eu is prevalent in Europe, TBEV-FE in the far-eastern region of Euroasia, while TBEV-Sib has been detected in almost all TBEV habitat areas [11]. At least eight WNV genetic lineages have been described, of which lineages 1 and 2 are most widely distributed [12]. Multiple WNV lineages have been isolated in Europe, with WNV-1a and WNV-2 being the main lineages responsible for human infections [13]. USUV strains are grouped within eight lineages based on their geographic origin of detection: Africa 1–3 and Europe 1–5 [14,15]. Although lineage 2 is dominant in human infections, USUV African lineages continue to be introduced in Europe [16].

Neuroinvasive clinical presentations differ according to the causative virus. TBEV-Eu usually causes biphasic disease with the first viremic and second meningoencephalitic phases, while TBEV-FE and TBEV-Sib show a monophasic course [17]. Neuroinvasive WNV disease is mainly presented as meningitis or encephalitis, whereas myelitis occurs in approximately 5–10% of cases [18]. In addition, some less frequent clinical presentations, such as polyradiculoneuritis, cerebellitis, and cauda equina arachnoiditis, are also reported [19,20]. The pathogenesis and clinical symptoms of USUV NID are similar to WNV [21], while TOSV presents mainly with meningitis (about 80% of cases) [22].

Numerous bird species have been shown to be infected with WNV and USUV. While American crows (*Corvus brachyrhynchos*) are highly susceptible to WNV infection, Eurasian blackbirds (*Turdus merula*) and great grey owls (*Strix nebulosa*) are highly vulnerable to USUV with high mortality rates [21]. Like in humans, flavivirus infections in horses are usually asymptomatic; however, few reports describe TBEV and WNV neuroinvasive diseases in horses [23,24].

A substantial TBE burden across endemic regions was indicated by a preliminary assessment at the end of the 2024 season. In 2024, the highest number of TBE cases was ever reported in Finland. Furthermore, the highest number in more than 20 years was documented in Lithuania. The increased number of cases compared to 2023 was observed in Norway and Germany [25]. In Europe, 19 countries reported human WNV infections in the 2024 transmission season. Compared to 137 regions in 2023, 212 regions reported locally acquired human cases of WNV infection in 2024, which represented the largest geographic distribution ever reported in a year. Additionally, the number of recorded cases exceeded the average monthly case number over the previous ten years. According to veterinary reports, there were 494 WNV outbreaks in horses and 447 in birds in Europe in 2024 [26].

In Croatia, TBEV and WNV are endemic in continental regions, while human USUV neuroinvasive infections are reported sporadically. Sporadic TOSV infections are also detected in residents of the Croatian littoral. TBEV is recorded continuously in northwestern and eastern counties with clustering in 2019 and 2022 in new micro-foci in the Gorski Kotar region, which divides the continental from the coastal part [27,28]. WNV infections occurred continuously from 2012 to 2019 and re-emerged in 2022 [29,30]. Sporadic human USUV neuroinvasive infections were detected during the 2013 and 2018 WNV outbreaks [31,32]. Only one symptomatic WNV neuroinvasive disease was reported in a horse during the 2022 Croatian outbreak [30], but asymptomatic acute infections were continuously detected [29,32]. Fatal WNV and USUV infections in birds were recorded in 2018 [32] and 2022 [30].

During the 2024 transmission season in Croatia, human neuroinvasive USUV infections re-emerged after a six-year absence, and a spread of WNV was observed. This study aimed to analyze the prevalence and molecular epidemiology of arbovirus infections detected in humans and animals in 2024. Additionally, the potential influence of climate on the occurrence of arboviral infections was assessed.

## 2. Materials and Methods

### 2.1. Patients with Neuroinvasive Disease Sampling

From April to November 2024, 154 hospitalized patients with NID were tested for the most common arboviruses reported in Croatia: TBEV, WNV, USUV, and TOSV. Both mosquito-borne (WNV; USUV) and tick-borne (TBEV) flaviviruses were included in testing due to the overlapping geographic distribution, similar clinical symptoms, and serological cross-reactivity. Patients were from 12/14 continental and 2/7 coastal Croatian counties. In all patients, cerebrospinal fluid (CSF), serum, and urine samples were collected in the acute phase of the disease. CSF and urine samples were tested for the viral RNA. Additionally, serum and CSF samples were tested for IgM and/or IgG antibodies. Diagnosis of arbovirus infection was confirmed according to the European Centre for Disease Control and Prevention (ECDC) criteria, which include: (a) virus isolation; (b) viral RNA in blood and/or CSF; (c) specific IgM antibody response in CSF; and/or (d) high IgM titer and IgG antibody detection confirmed by a virus neutralization test (VNT) [33]. Urine was also tested due to its noninvasive collection and high viral load since several recently published articles demonstrated the diagnostic utility of urine reverse-transcription polymerase chain reaction (RT-qPCR) for WNV NID confirmation and its superiority over CSF testing [34,35].

### 2.2. Horse and Bird Sampling

Horse serum samples were collected from March to November as part of the WNV surveillance program. A total of 1168 samples were tested to determine the WNV IgG seroprevalence. For screening of acute asymptomatic infections (IgM seropositive), 395 randomly selected samples were tested. Horses were from 20/21 Croatian counties. All horses were asymptomatic at the sampling time, had not received the WNV vaccine, and were not moved in international transport or between counties. For TBEV seroprevalence determination, 428 additional horse samples from 19/21 Croatian counties were tested for TBEV IgG antibodies. A total of 69 dead wild birds collected during 2024 were tested for WNV and USUV as part of the WNV surveillance program. The birds belonged to the orders Anseriformes (*n* = 28), Passeriformes (*n* = 23), Ciconiiformes (*n* = 8), Pelecaniformes (*n* = 5), Columbiformes (*n* = 4), and Galliformes (*n* = 1).

### 2.3. Mosquito Sampling

CDC Mini Light traps (BioQuip Products, Rancho Dominguez, CA, USA) equipped with dry ice (CO_2_) as an attractant were used to collect mosquitoes. Traps were placed approximately 1.5 m from the ground and set in the late afternoon before sunset, left overnight, and removed after sunrise (07:00–10:00). Traps with CO_2_ were set from May to October every 14 days at the same eight collection sites. A total of 96 sampling occasions were gathered. The following habitats were chosen for sampling: the woods (one location), a populated area close to the woods (six locations), and a meadow near a stable with horses (one location).

The sampled mosquitoes were transported to the laboratory in containers with dry ice, transferred to plastic containers, and stored on dry ice until identification. Female mosquitoes were morphologically identified by species or species complex on a chilling surface under a stereomicroscope using the determination key by Becker et al. (2020) [36]. Specimens belonging to the same species/complex collected on the same day and at the same sampling site were pooled, with up to 60 individuals per pool, and stored at −80 °C until virological testing. Mosquito pools were tested for WNV and USUV RNA.

### 2.4. Molecular Diagnostics and Sequencing

A High Pure Viral Nucleic Acid Kit (Roche Applied Science, Penzberg, Germany) was used to extract viral RNA. Viral RNA was detected using Taqman real-time specific RT-qPCR assays according to protocols: TBEV (Scwaiger and Casinotti, 2003) [37], WNV (Tang et al., 2006) [38], USUV (Nikolay et al., 2015) [39], and TOSV (Weidmann et al., 2008) [40]. Two WNV strains detected by RT-qPCR (from the urine of a patient with the NID and a bird brain) and three USUV strains (from CSF of patients with NID) were further subjected to conventional RT-PCR according to the protocol by Weissenbock et al. (2002) [41].

The same primers were used for RT-PCR amplification product Sanger sequencing. The obtained sequences were genotyped and phylogenetically grouped based on a comparison with strains retrieved from GenBank and obtained using the BLAST algorithm (http://www.ncbi.nlm.nih.gov, accessed on 12 August 2024). MEGA11 was used for a maximum likelihood phylogenetic analysis and the evolutionary analyses [42].

### 2.5. Serology

Detection of arbovirus IgM and/or IgG antibodies in human samples was performed using either an enzyme-linked immunosorbent assay (ELISA; TBEV, WNV, USUV) or an indirect immunofluorescence assay (IFA; TOSV) (Table 1). TBEV- and WNV IgM- and IgG-positive samples were additionally tested for IgG avidity (ELISA) to confirm recent infection. Furthermore, samples with detected cross-reactive flavivirus antibodies were confirmed using a virus neutralization test (VNT) validated by the WOAH Reference Laboratory for West Nile Fever, Istituto Zooprofilattico Sperimentale G. Caporale, Teramo, Italy [43]. The TBEV Ljubljana strain (provided by the European Virus Archive goes Global project), WNV lineage 2 strain isolated from a goshawk, and USUV Europe 2 lineage strain isolated from a blackbird were used as antigens for the VNT. The Reed and Muench formula was used to determine the virus titer (TCID_50_). After inactivation (56 °C for 30 min), 25 μL of serial two-fold serum dilutions starting at 1:5 was prepared. A volume of 25 μL containing 100 TCID_50_ of the virus was added to each microtiter plate well, and mixtures were incubated at 37 °C with CO_2_ for 1 h. In the final step, 50 μL of 2 × 10^5^ Vero E6 cells/mL in DMEM with 5% heat-inactivated fetal calf serum was added. The plates were incubated at 37 °C with CO_2_ for five days and checked for the cytopathic effect starting from the third day. The neutralizing antibody titer ≥10 was considered a positive result [27].

Horse serum samples were tested for WNV IgM and/or IgG antibodies using a competitive ELISA (INgezim West Nile virus IgM and IgG, Gold Standard Diagnostics, Madrid, Spain). For the detection of TBEV IgG antibodies, a commercial ELISA (Immunozym FSME (TBE) IgG All Species; PROGEN Biotechnik GmbH, Heidelberg, Germany) was used. Samples initially reactive for IgG antibodies were confirmed using a VNT.

### 2.6. Climate Data Collection and Analysis

The climate assessment included analyses of mean air temperature, humidity, and precipitation for 2024, expressed as percentiles for the 1991–2020 multiannual average [44,45]. Monthly mean air temperatures (°C), relative air humidity (%), and total precipitation (overall rainfall in mm) data for eleven counties with recorded acute WNV and USUV infections (humans, horses, and birds) were provided by the Croatian Meteorological and Hydrological Service [45]. The correlation between the overall (human, horse, bird) number of arboviral infections (N) and the mean monthly weather conditions was tested with the Pearson’s correlation coefficient (r, *p* < 0.05) using the Statistica Version 14.1.0.8 TIBCO Software Inc. (San Ramon, CA, USA). The size of correlation was interpreted as follows: 0.90 to 1.00 (−0.90 to −1.00) very high positive (negative); 0.70 to 0.890 (−0.70 to −0.89) high positive (negative); 0.50–0.69 (−0.50 to −0.69) moderate positive (negative); 0.30 to 0.49 (0.30 to −0.49) low positive (negative); and 0.00 to 0.29 (0.00 to −0.29) little if any positive (negative) correlation [46].

## 3. Results

### 3.1. Arboviral Infections in Patients with Neuroinvasive Diseases, Horses, and Birds

Arboviral etiology was confirmed in 33 (21.42%) patients with NID. WNV infections were most prevalent (17; 11.03%). TBEV was detected in 10 (6.49%), USUV in 5 (3.24%) of patients, and TOSV in one patient (0.64%). Three patients were TBEV IgG seropositive, one was WNV, and one was USUV IgG positive (Table 2). Nine of ten patients with acute TBE reported a tick bite.

The results of virology testing are presented in Table 3. TBEV infection was confirmed in all patients by detection of IgM antibodies in CSF samples. WNV NID was confirmed by the IgM detection in CSF samples in 91.66% of samples. Five urine samples were RT-qPCR positive. USUV infection was confirmed in four patients by detection of USUV RNA in the CSF sample and detection of USUV antibodies in the serum sample (positive ELISA confirmed by VNT) in one patient. TOSV infection was confirmed by the seroconversion in paired serum samples using IFA (I sample—IgM positive; II sample—IgM/IgG positive). One WNV strain detected in urine and three USUV strains detected in CSF were sequenced.

Five samples showed cross-reactive flavivirus antibodies (Table 4). In one sample (No. 1), TBEV infection was confirmed by the detection of IgM/IgG antibodies in a serum sample, which was confirmed by the detection of TBEV neutralizing antibodies. In two samples (No. 2 and 3), WNV RNA was detected in urine samples.

**Figure 1:**
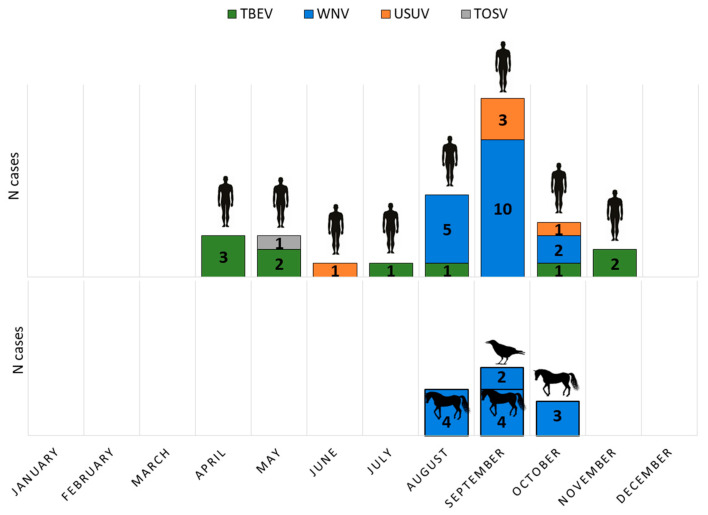
Seasonal distribution of arboviral infections in humans, horses, and birds.

Human TBEV infections showed a bimodal seasonality, with a larger peak from April to August and a smaller one in October and November. WNV infections occurred from August to October. USUV was detected in June (one case), September (3 cases), and October (one case), while TOSV infection was confirmed at the end of May (Figure 1).

Acute asymptomatic WNV infections (IgM positive) were detected in 11/395 (2.78%) of horses during August, September, and October. Two WNV-positive dead crows (*Corvus corone*) were found in September (Figure 1).

**Figure 2:**
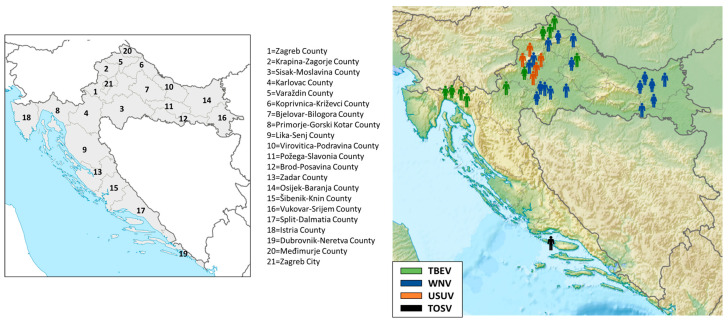
Geographic distribution of human neuroinvasive arboviral infections, 2024.

The geographic distribution of acute cases is presented in Figure 2. All but one patient (TOSV infection; Split-Dalmatia County, No. 17) were residents of continental Croatian counties. TBEV infections were recorded in five northwestern continental counties (No. 1, 4, 5, 7, and 20), with a small clustering of four cases in the Gorski Kotar region (Primorje-Gorski Kotar County, No. 8). WNV infections were recorded in six counties: Sisak-Moslavina (No. 3), Osijek-Baranja (No. 14), Koprivnica-Križevci (No. 6), Brod-Posavina (No. 12), Varaždin (No. 5), and the City of Zagreb (No. 21), and for the first time in Bjelovar-Bilogora County (No. 7). USUV infections were detected in three counties (No. 1, 2, and 21).

**Figure 3:**
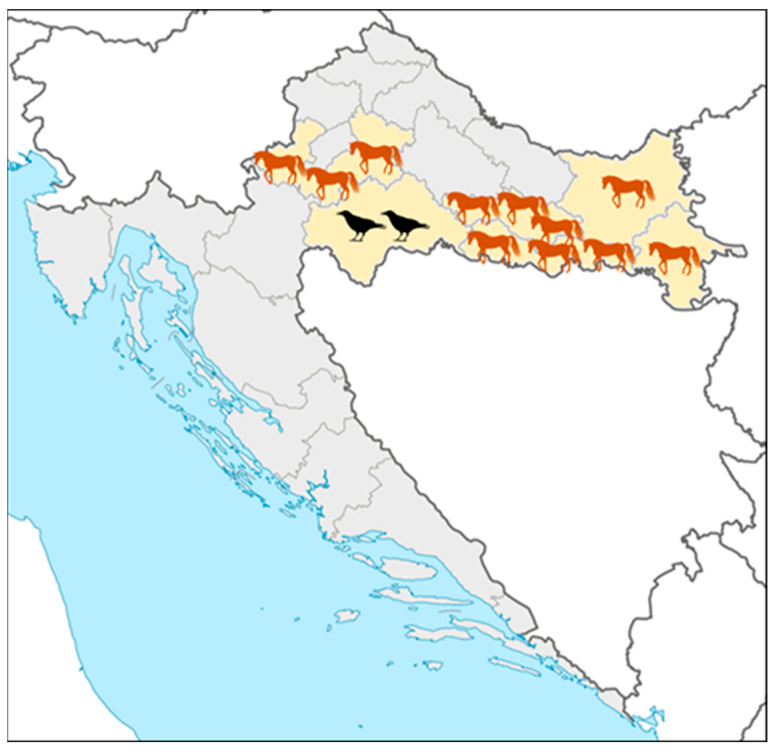
Geographic distribution of acute West Nile virus infections in horses and birds.

Acute asymptomatic WNV infections in horses were recorded in five continental counties (Figure 3): Zagreb County, Požega-Slavonia, Brod-Posavina, Osijek-Baranja, and Vukovar County (No. 1, 11, 12, 14, and 16; Figure 3). Fatal WNV infections in birds were reported in Sisak-Moslavina County (No. 3; Figure 3).

### 3.2. Flavivirus Seroprevalence in Horses

**Figure 4:**
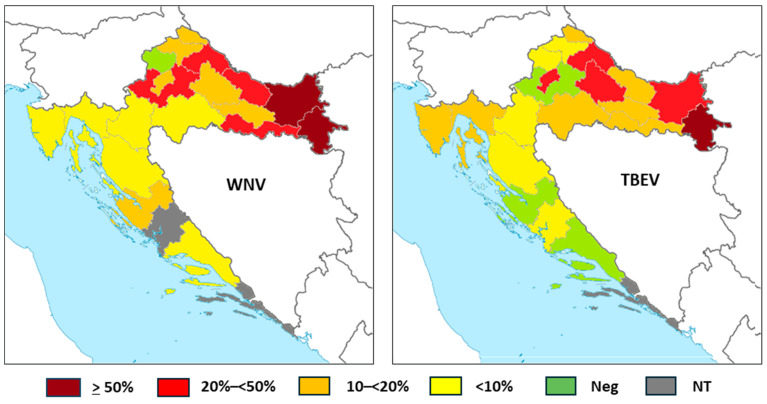
Tick-borne encephalitis virus IgG seroprevalence rates in horses according to the geographic region (NT = not tested).

The WNV and TBEV IgG seroprevalence in horses according to counties is presented in Table 5 and Figure 4.

WNV IgG antibodies were detected in 276/1168 (23.63%; 95%CI = 21.22–26.17) horses. The seropositivity was higher in continental counties, ranging from 5.26% (95%CI = 0.13–26.03) in Karlovac County to 54.79% (95%CI = 42.70–66.48) in Vukovar-Srijem County. No seropositive horses were detected in Krapina-Zagorje County. In coastal counties, the lowest seropositivity was observed in Primorje-Gorski Kotar County (4.55%; 95%CI = 0.95–12.71) and the highest in Zadar County (16.67%, 95%CI = 0.42–64.12). In Zagreb County, Zadar County, and Split-Dalmatia County, all horses were seronegative.

TBEV IgG antibodies were found in 68/428 (15.88%; 95%CI = 12.55–19.70) horses. Similarly to WNV, higher seropositivity was observed in continental counties, from 5.00% (95%CI = 0.13–24.87) in Krapina-Zagorje to 72.73% (95%CI = 49.78–89.27) in Vukovar-Srijem County. In coastal counties, the seroprevalence ranged from 3.92% (95%CI = 0.48–13.46) in Lika-Senj County to 12.82% (95%CI = 4.30–27.43) in Primorje-Gorski Kotar County. In two coastal counties, no TBEV seropositive horses were detected.

### 3.3. Mosquito Species Detected and Tested

In 2024, a total of 7726 female mosquitoes were collected and identified in the City of Zagreb during the entomological survey.

The collected mosquitoes belong to 12 species. Of these, 66.68% of mosquitoes were identified as *Aedes vexans*, followed by *Ae. sticticus* (20.14%), *Ae. rusticus* (4.96%), *Ae. albopictus* (3.70%), and *Culex pipiens* complex (1.66%). The remaining seven species were represented by less than 1%. Mosquito specimens were sorted into 311 pools and tested for the presence of WNV and USUV RNA (Table 6). No WNV- and USUV RNA-positive pool was detected.

### 3.4. Molecular Epidemiology of Arboviral Infections in Humans and Animals

**Figure 5:**
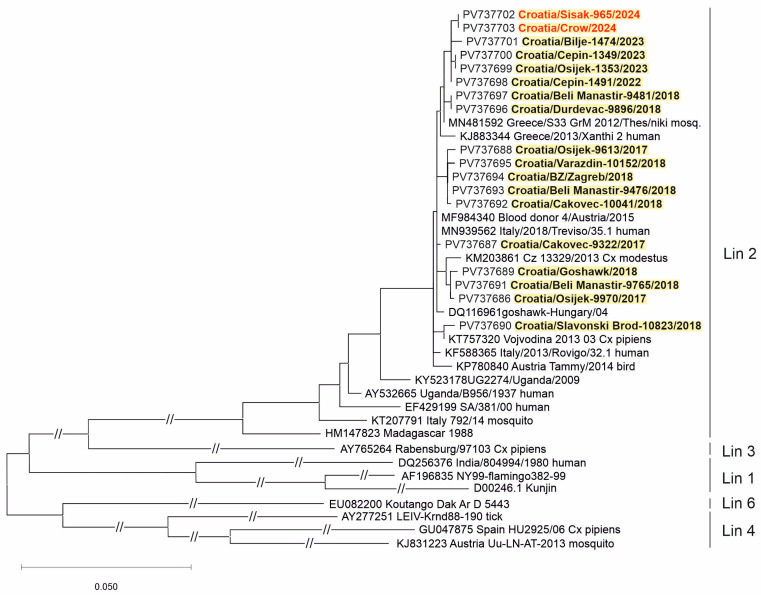
A phylogenetic tree of the West Nile virus. The evolutionary history was inferred on 794 positions of the NS5 gene. The scale bar indicates nucleotide substitutions per site. WNV isolates from Croatia sequenced in previous transmission seasons [30,31,32] are bold, and those sequenced in 2024 are marked in red and bold. The WNV lineages proposed by Rizzoli et al. (2015) [12] are denoted on the right.

**Figure 6:**
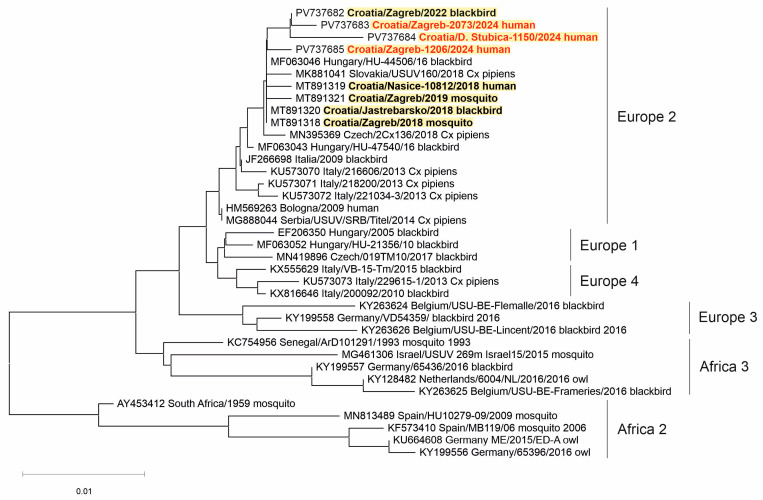
A phylogenetic tree of the Usutu virus. The evolutionary history was inferred on 495 positions of the NS5 gene. The scale bar indicates nucleotide substitutions per site. USUV isolates from Croatia sequenced in previous transmission seasons [30,32] are bold, and those sequenced in 2024 are marked in red and bold. The USUV lineages proposed by Cadar et al. (2017) [14] are denoted on the right.

Two WNV strains (from a urine sample of a patient with NID and a brain tissue of a dead crow) and three USUV strains (from the CSF samples of patients with NID) were sequenced. Phylogenetic analysis of the detected viruses revealed WNV lineage 2 (Figure 5) and USUV Europe 2 lineage (Figure 6).

### 3.5. Impact of Climate and Meteorological Conditions in 2024 on Arboviral Infections Occurrence

Since WNV and USUV are closely related and ecologically similar viruses, the impact of climate was analyzed for both viruses in two biogeographic regions: Continental and Alpine [47]. According to the percentile distribution [45], 2024 was an extremely warm year throughout Croatia, and the annual precipitation in most of Croatia’s counties was within normal limits when compared to the 30-year reference climate period 1991–2020 [44]. Deviations of the mean air temperature ranged from 1.3 °C to 2.7 °C, and precipitation deviations ranged from 82.9% to 124.4%. Rainy conditions prevailed in the central part of the Alpine biogeographic region (Lika-Senj County) and the northwestern part of the Continental region (Zagreb City). Dry conditions prevailed in the northern part of the Continental region (Koprivnica-Križevci and Bjelovar-Bilogora County) and the southern part of the Mediterranean region (Split-Dalmatia County). A significant positive correlation (*p* < 0.05) was found between the mean total precipitation and the number of arboviral infections (human, horse, and bird) in Zagreb City, Zagreb County, Koprivnica-Križevci, Bjelovar-Bilogora, and Varaždin County (Table 7). Pearson’s correlation coefficient observed between mean monthly air temperatures (°C) and the overall number of WNV and USUV infections varied from r = −0.0413 to r = +0.4849 between counties, but without statistical significance. In addition, no significant difference was observed between monthly relative air humidity (%) and the overall number of WNV/USUV infections (Pearson’s correlation coefficient r = −0.4535 to r = +0.3033).

## 4. Discussion

The number of cases and incidence rates of TBE in Europe vary every year. At the end of the 2024 TBE season, preliminary data from 12 European countries (Scandinavia and Baltic countries and Central Europe) indicated a high disease burden in endemic regions. In Norway, the number of cases has increased compared to 2022 and 2023, while in Finland, 2024 was a year with the highest number of TBE cases ever reported. In Germany and Switzerland, the number of TBE cases in 2024 was the second highest after the year 2020. A consistently high TBE burden was also observed in northeastern Italy [25].

In Croatia, the number of TBE cases in the past 10 years ranged from 4 to 25. In 2024, 10 TBEV patients were reported, which was within the range of previous years. The seasonal distribution of cases (April–November) was also similar to previous years (2017–2023), showing two peaks (April–May and October–November) [28]. Likewise, between 2012 and 2020, year-round TBE transmission was observed in Europe, with most cases (98.8%) occurring between April and November. In all the years analyzed, except for 2012 and 2016, the distribution of autochthonous cases showed a bimodal pattern, with a first major peak around the first week of July and a smaller secondary peak occurring at the end of September [48]. However, a large Croatian study conducted from 2002 to 2018 showed a seasonal pattern of TBE peaking in June and July [49].

The WNV transmission season in Europe was quite intense in 2024 (1436 locally acquired cases as of 4 December 2024), with Italy and Greece reporting the highest number of cases (455 and 217, respectively) [26]. Similarly, in Croatia, the number of hospitalized patients with WNV NID in 2024 (17 cases) was higher than in 2023 (10 cases) and 2022 (6 cases) [30], representing the second most intense season after 2018 (54 cases) [32].

Analyzing the seasonal distribution of WNV in European countries, the earliest and latest months of WNV onset were, respectively, March 2024 (Spain) and November 2024 (Germany) [26]. A remarkable increase in WNF cases in the early summer of 2024 was observed in Israel [50]. In Croatia, human WNV infections were detected from August to October.

Like in previous transmission seasons in Croatia, acute WNV infections were confirmed in animals as well. From August to October, acute asymptomatic infections were confirmed by the IgM antibody detection in 11 horses. Additionally, WNV was confirmed in the brain tissue of two crows found dead in September. In 2024, WNV outbreaks among equids and birds were also reported in European countries. However, the WNV transmission season in Europe has extended, with the earliest and latest dates of bird and/or equid outbreaks on 2 April and 25 November 2024 [26].

In Europe, most human USUV infections have been reported in Italy [51]. In Croatia, human neuroinvasive USUV infections re-emerged in the 2024 transmission season after a six-year absence. Five patients with NID were recorded in three northwestern counties. Sporadic USUV infections were previously detected during the WNV outbreaks in 2013 [31] and 2018 [32].

The combined impact of various climatic factors across different seasons may affect the intensity of arbovirus circulation and transmission peaks by influencing mosquito populations, viral replication, and host–vector interactions. There are many ecological factors sustaining the dynamics of WNV and USUV prevalence, like the mosquito–bird–mosquito transmission cycle maintaining WNV infections in nature [52], different weather-dependent factors that impact the biology of mosquitoes (e.g., changes in reproduction, population size) [53], and the epidemic oscillations associated with seasonal change (extrinsic) and dynamic interaction between host and vector populations (intrinsic).

According to the World Meteorological Organization’s State of the Global Climate 2024 report, 2024 was the warmest in the 175-year observational record, with the global mean temperature rising to 1.55 ± 0.13 °C above the 1850–1900 average [54]. In all Croatian counties, climate conditions were classified as extremely warm, according to the percentile distribution [45], concerning the corresponding 30-year reference climate period 1991–2020 [44]. With 97.1% of WNV and USUV cases (in humans, horses, and birds) reported between August and October 2024 in Croatia, the results of this study align with previous findings regarding the annual peak prevalence of the disease, which occurs between late July and early September [55].

August was extremely warm for most of Croatia. At most weather stations, it was the warmest month ever recorded since monitoring started. September was a warm month throughout Continental Croatia and in most of the Alpine region, while October was a warm to very warm month in the Continental region. In contrast, the annual precipitation in the majority of Croatia’s counties in 2024 was within normal limits when compared to the same 30-year reference climatic period 1991–2020 [44,45]. According to the percentile distribution, normal precipitation conditions prevailed in most of Croatia during August, while in northern and eastern parts of Continental Croatia, August was dry and even extremely dry. During September, rainy conditions prevailed in most of the Continental and Alpine regions, while in October, monthly precipitation across most of inland Croatia remained within normal levels. A significant positive correlation was observed between the number of arboviral infections (human, horse, bird) and the mean total precipitation (mm) in Zagreb City, Zagreb County, Koprivnica-Križevci County, Bjelovar-Bilogora, and Varaždin County. The highest Pearson’s correlation coefficient was recorded in Bjelovar-Bilogora County, where human WNV infections were reported for the first time. Although not statistically significant, a moderate positive correlation was observed between mean monthly air temperatures and the total number of WNV infections in Brod-Posavina County. In contrast, a moderate negative correlation was noted between monthly relative air humidity and the number of WNV infections in the same region. Additionally, Varaždin County exhibited a moderate positive correlation between monthly air temperature and the number of WNV cases. Although the small number of cases in several counties likely influenced the correlation results, the detected coefficients suggested notable relationships between the variables, aligning with previous findings on the impact of weather conditions on pathogen circulation.

A study conducted in Serbia revealed that spring temperatures had a significant influence on the WNV epidemiology, highlighting the importance of spring conditions for enhancing WNV circulation [56]. A similar correlation was observed at a European level, but with summertime conditions. It appears that only summer temperatures influence WNV incidence peaks, with infection peaks occurring earlier when summer temperatures are higher [57]. Warmer conditions may increase the mosquito biting rate, host-to-vector transmission, and shorten the extrinsic incubation period in mosquitoes [58].

The interesting results were observed in Greece, one of the European countries with the highest annual WNV incidence. A warmer winter, a warmer, drier spring, and a more humid, though not necessarily warmer, summer were linked to two more severe Greek WNV outbreaks (2010 and 2018). These findings suggested that global warming may facilitate vector hibernation. In addition, warm, drier springs may accelerate the WNV transmission, leading to a higher circulation in the months that follow, while higher summer humidity contributes to the mosquito-borne disease peaking, most likely by accelerating vector reproduction [59].

In this study, serologic evidence of WNV infection was observed in 23.63% of horses, with the highest seropositivity up to 54.79% in regions with confirmed human cases. Furthermore, IgG seropositive horses (4.55–16.67%) were also detected in the coastal regions, although there have not been any reports of human WNV infections at the Croatian littoral so far. The TBEV IgG seropositivity was 15.88%, with the highest seroprevalence up to 72.73% in eastern and northwestern regions previously known as high-risk areas for TBEV [28].

Similar to previous seasons [29,30,32], WNV lineage 2 was confirmed in a human and a crow in 2024 in Croatia. WNV lineage 2a was responsible for most cases in Europe in the period 2016–2019 [60]. Co-circulation of WNV lineages 1 and 2 was noted in Italy in 2022, whereas only WNV lineage 2 circulation was documented in 2018 [61].

Three USUV strains detected in the CSF samples of Croatian patients in 2024 clustered within the USUV Europe 2 lineage. The Europe 2 lineage was most frequently detected in humans (Austria, Italy, Croatia). However, in Italy, the USUV Europe 1 lineage was more prevalent. In addition, the USUV Africa 3 lineage has been sporadically detected in human infections in Austria [14].

In 2024, no WNV or USUV RNA was detected in mosquitoes in Croatia. Previous entomological studies have shown the presence of WNV lineage 2 in the *Culex pipiens* complex (2023) [62] and USUV Europe 2 lineage in the *Cx. pipiens* complex (2018, 2019) [63]. Of the total eight habitats chosen for sampling mosquitoes, one was a woodland, six locations were populated areas close to the woods, and one location was a meadow near a stable with horses. The nature of the locations resulted in the flood mosquito species *Aedes vexans* and *Aedes sticticus* being the most abundant mosquito species collected in 2024.

TOSV is a frequent cause of CNS infections in some endemic regions. In Tuscany (central Italy), at least 80% of summertime CNS viral infections in children are caused by TOSV [64]. In addition, an increasing trend of neuroinvasive TOSV infections over time was observed in adults in Italy [65]. In Croatia, sporadic TOSV NIDs are reported continuously in inhabitants of the Croatian littoral [66]. In 2024, TOSV was confirmed in one patient who developed symptoms at the end of May. In Mediterranean countries, sandfly activity is seasonal, occurring from May to October, with peak activity observed in July and August [9,59]. The sandfly seasonality and high temperatures in 2024 explain the early detection of TOSV infection in Croatia. TOSV RNA was not detected in the patient with NID in 2024; however, previous studies have shown co-circulation of TOSV lineages B and C in Croatia [67].

This study has some limitations that need to be addressed. In some counties, a low number of horse samples was collected, which may influence the seroprevalence results. In addition, mosquito sampling was conducted in a limited area in northwestern Croatia (the City of Zagreb) with a small number of collected *Cx. pipiens* complex mosquitoes, the main vectors of WNV and USUV (1.66% of the total number), which had an impact on the negative mosquito testing results.

A prospective observational study for arbovirus preparedness in Southeast Europe (MERMAIDS-ARBO) has highlighted the need to strengthen routine diagnostics to improve clinical diagnostics in risk areas. Therefore, testing for arboviral infections should be included in the routine diagnostic protocols in all patients presenting with arbovirus-compatible symptoms during the transmission season [68]. Furthermore, enhancing arbovirus surveillance systems is crucial for the early detection of emerging and re-emerging outbreaks. This includes investing in efforts to raise awareness of arboviral infections among clinicians, improving access to specialized diagnostic tools, and advancing the development of effective, accessible vaccines and treatments [60].

## 5. Conclusions

The 2024 season was the second most intensive arbovirus transmission season in Croatia since 2012. Neuroinvasive flavivirus infections were reported in continental Croatian regions, with sporadic detection of TOSV along the Croatian littoral. The spreading of WNV and the re-emergence of human USUV infections, as well as the detection of acute WNV infections in birds and horses, highlight the need for continuous arbovirus surveillance. Furthermore, mosquito monitoring should be expanded to all continental counties, especially regions with a high incidence of human and animal infections.

## Figures and Tables

**Table 1:** Serology methods used for the detection of arboviral infections in humans.

Virus	Serology Method	Kit Name
TBEV	IgM/IgG ELISA, IgG avidity, VNT (*in-house*)	Anti-TBEV IgM ELISA; Anti-TBEV IgG ELISA 2.0; TBEV-Avidity ELISA (IgG); Euroimmun, Lübeck, Germany
WNV	IgM/IgG ELISA, IgG avidity, VNT (*in-house*)	Anti-WNV IgM ELISA; Anti-WNV IgG ELISA; WNV-Avidity ELISA (IgG); Euroimmun, Lübeck, Germany
USUV	IgG ELISA, VNT (*in-house*)	Anti-USUV IgG ELISA; Euroimmun, Lübeck, Germany
TOSV	IgM/IgG IFA	Sandfly Fever Mosaic IIF; Euroimmun, Lübeck, Germany

TBEV = tick-borne encephalitis virus; WNV = West Nile virus; USUV = Usutu virus; TOSV = Toscana virus; ELISA = enzyme-linked immunosorbent assay; IFA = indirect immunofluorescence assay; VNT = virus neutralization test.

**Table 2:** Arboviruses detected in hospitalized patients with neuroinvasive diseases.

Virus	Acute Infections; N/%	IgG Seropositive; N/% (95%CI)
TBEV	10/6.49	3/1.95 (0.40–5.59)
WNV	17/11.03	1/0.65 (0.02–3.56)
USUV	5/3.24	1/0.65 (002–3.56)
TOSV	1/0.64	0/0 (0–2.37) *

TBEV = tick-borne encephalitis virus, WNV = West Nile virus; USUV = Usutu virus, TOSV = Toscana virus, CI = confidence interval; * one-sided 97.5% confidence interval.

**Table 3:** Results of serology and RT-PCR testing in patients with neuroinvasive arbovirus infections.

Detected Virus	Serum ELISA N (%) Positive		CSF ELISA N (%) Positive		RT-qPCR N (%) Positive		Sequencing N
	IgM	IgG	IgM	IgG	CSF	Urine	
TBEV (*n* = 10)	10 (100)	9 (90.00)	10 (100)	8 (80.00)	0 (0)	0 (0)	0
WNV (*n* = 17)	12 (70.58)	10 (58.82)	11 (91.66)	3 (17.64)	0 (0)	5 (29.41)	1
USUV (*n* = 5)	NT	2 (NA)	NT	0 (NA)	4 (NA)	2 (NA)	3

CSF = cerebrospinal fluid, NT = not tested, NA = not applicable, TBEV = tick-borne encephalitis virus, WNV = West Nile virus, USUV = Usutu virus.

**Table 4:** Results of samples with cross-reactive serum flavivirus antibodies.

Sample No.	ELISA TBEV		ELISA WNV		ELISA USUV		RT-qPCR CSF/Urine	Serum VNT Titer
	Serum IgM/IgG	CSF IgM/IgG	Serum IgM/IgG	CSF IgM/IgG	Serum IgG	CSF IgG		
1	Positive/ Positive	Negative/ Positive	Negative/ Positive	Negative/ Positive	Negative	Negative	Negative/ Negative	TBEV 20
2	Negative/ Positive	Negative/ Negative	Positive/ Positive	Positive/ Negative	Negative	Negative	WNV urine positive	NT
3	Negative/ Positive	Negative/ Negative	Positive/ Positive	Negative/ Positive	Negative	Negative	WNV urine positive	NT
4	Negative/ Positive	Negative/ Negative	Positive/ Positive	Negative/ Positive	Positive	Negative	Negative/ Negative	WNV 10
5	Negative/ Negative	Negative/ Negative	Positive/ Negative	Negative/ Negative	Positive	Negative	Negative/ Negative	USUV 40

**Table 5:** West Nile virus and tick-borne encephalitis virus IgG seroprevalence in horses by counties.

County	WNV IgG			TBEV IgG		
	N	N (%) Positive	95%CI	N	N (%) Positive	95%CI
Zagreb	115	24 (20.87)	13.85–29.44	9	0 (0)	0–33.63 *
Krapina-Zagorje	22	0 (0)	0–15.44 *	20	1 (5.00)	0.13–24.87
Sisak-Moslavina	26	2 (7.69)	0.95–25.13	19	3 (15.79)	3.38–39.58
Karlovac	19	1 (5.26)	0.13–26.03	15	1 (6.67)	0.17–31.95
Varaždin	66	10 (15.15)	7.51–26.10	11	1 (9.09)	0.23–41.28
Koprivnica-Križevci	33	7 (21.21)	8.98–38.91	11	3 (27.27)	6.02–60.97
Bjelovar-Bilogora	72	9 (12.50)	5.88–22.41	17	4 (23.53)	6.81–49.90
Primorje-Gorski Kotar	66	3 (4.55)	0.95–12.71	39	5 (12.82)	4.30–27.43
Lika-Senj	22	2 (9.09)	1.12–29.16	51	2 (3.92)	0.48–13.46
Virovitica-Podravina	95	21 (22.11)	14.23–31.78	20	3 (15.00)	0.21–37.89
Požega-Slavonia	46	7 (15.22)	6.34–28.87	16	3 (18.75)	4.05–45.65
Brod-Posavina	86	20 (23.3)	14.8–33.6	21	3 (14.3)	3.1–36.3
Zadar	6	1 (NA)	NA	19	0 (0)	0–17.65 *
Osijek-Baranja	202	101 (50.00)	42.90–57.10	21	7 (33.33)	14.59–56.97
Šibenik-Knin	NT	NA	NA	20	1 (5.00)	0.13–24.87
Vukovar-Srijem	73	40 (54.79)	42.7–66.5	22	16 (72.73)	49.8–89.3
Split-Dalmatia	16	1 (6.25)	0.16–30.23	28	0 (0)	0–12.34 *
Istria	45	3 (6.67)	1.40–18.27	35	4 (11.43)	3.20–26.74
Dubrovnik-Neretva	NT	NA	NA	NT	NA	NA
Međimurje	53	7 (13.21)	5.48–25.34	13	2 (15.38)	1.92–45.45
Zagreb City	105	17 (16.19)	9.72–24.65	21	9 (42.86)	21.82–65.98

NA = not applicable; NT = not tested; * one-sided 97.5% confidence interval.

**Table 6:** Number of mosquitoes by species prepared in pools for testing for the presence of West Nile virus and Usutu virus RNA.

Mosquito Species	Total (%)	Number of Mosquito Pools
*Anopheles claviger*	2 (0.03)	1
*Anopheles plumbeus*	55 (0.71)	11
*Aedes albopictus*	286 (3.70)	44
*Aedes cantans*	53 (0.69)	4
*Aedes cinereus*	35 (0.45)	4
*Aedes geniculatus*	49 (0.63)	6
*Aedes rossicus*	3 (0.04)	1
*Aedes rusticus*	383 (4.96)	12
*Aedes sticticus*	1556 (20.14)	53
*Aedes vexans*	5152 (66.68)	126
*Coquillettidia richiardii*	24 (0.31)	2
*Culex pipiens complex*	128 (1.66)	47
Total	7726 (100)	311

**Table 7:** Correlation between climate parameters and arboviral infections recorded in eleven counties in Continental and Alpine biogeographic region in Croatia.

County	Temperature (°C)		Relative Humidity (%)		Total Precipitation (mm)	
	*r*	*p*	*r*	*p*	*r*	*p*
Zagreb City	0.2849	0.3694	0.0811	0.8021	0.6367	0.0260
Zagreb County	0.2897	0.3610	0.0356	0.9125	0.6493	0.0223
Sisak-Moslavina	0.4032	0.1938	−0.1592	0.6211	0.3985	0.1995
Osijek-Baranja	0.2486	0.4359	−0.1403	0.6637	0.4659	0.1269
Brod-Posavina	0.4849	0.1101	−0.4535	0.1386	−0.1763	0.5836
Koprivnica-Križevci	0.1474	0.6477	−0.1270	0.6941	0.6192	0.0318
Krapina-Zagorje	0.1493	0.6433	0.0557	0.8636	0.5533	0.0620
Bjelovar-Bilogora	0.1477	0.6468	0.0059	0.9855	0.8496	0.0005
Varaždin	0.1098	0.7341	0.3033	0.3378	0.6076	0.0361
Požega-Slavonia	0.1839	0.5673	0.0466	0.8855	−0.0546	0.8662
Vukovar-Srijem	−0.0413	0.8985	0.2248	0.4823	−0.0330	0.9190

## Data Availability

The original contributions presented in the study are included in the article, further inquiries can be directed to the corresponding author.
